# Deep-Sea Marine Metabolites as Promising Anti-Tubercular Agents: CADD-Guided Targeting of the F_420_-Dependent Oxidoreductase

**DOI:** 10.3390/md24020058

**Published:** 2026-01-31

**Authors:** Ria Desai, Amane A. Alaroud, Gagan Preet, Rishi Vachaspathy Astakala, Rainer Ebel, Marcel Jaspars

**Affiliations:** 1 Marine Biodiscovery Centre, Department of Chemistry, University of Aberdeen, Aberdeen AB24 3UE, UK; ria.desai@abdn.ac.uk (R.D.); gagan.preet1@abdn.ac.uk (G.P.); rishi.astakala1@abdn.ac.uk (R.V.A.); r.ebel@abdn.ac.uk (R.E.); 2 Honorary Research Fellow, Rowett Institute, University of Aberdeen, Aberdeen AB25 2ZD, UK; amane.alaroud@abdn.ac.uk; 3 Department of Pharmaceutics and Pharmaceutical Biotechnology, Faculty of Pharmacy, Mutah University, Karak 61710, Jordan

**Keywords:** *Mycobacterium tuberculosis*, F_420_-dependent oxidoreductase (Rv1155), deep-sea metabolites, drug discovery, BBNJ

## Abstract

Tuberculosis, caused by *Mycobacterium tuberculosis* (M. tb), remains a leading global threat, escalated now by the rise of multidrug-resistant (MDR-TB) and extensively drug-resistant (XDR-TB) strains. In search of a novel anti-tubercular agent with a distinct mechanism of action, this study explores deep-sea marine metabolites as potential inhibitors of the F_420_-dependent oxidoreductase Rv1155, a redox enzyme essential for M. tb survival. A total of 2773 marine-derived compounds curated from the CMNPD, Reaxys, and MarinLit databases were screened using an integrated CADD workflow combining molecular docking, in-silico ADMET profiling, and molecular dynamics (MD) simulations. Docking identified 68 metabolites with strong affinity (−10.98 to −15.95 kcal/mol) for the Rv1155 binding pocket, and from which three compounds, Upenamide (CMNPD_22964), Aspyronol (Compound_1749), and Fiscpropionate F (Compound_1796), were shortlisted as hit candidates. Among these, Upenamide displayed the strongest binding (Δ*G* = −28.56 kcal/mol) with stable RMSD and hydrogen bond persistence during 100 ns MD simulation, while Aspyronol demonstrated a promising ADMET profile comparable to the native cofactor F_4202_. MM-GBSA analysis further confirmed the strong binding strength (Δ*G* _bind = −24.77 to −34.07 kcal/mol) for all three hit candidates. These findings confirm the strong and stable interaction of selected deep-sea marine metabolites with Rv1155. This validated screening pipeline established here provides a cost-effective framework for future experimental validation and expansion to additional F_420_-related drug targets in M. tb.

## 1. Introduction

Tuberculosis (TB), caused by *Mycobacterium tuberculosis* (M. tb), continues to be a global health burden and remains the leading cause of death from a single infectious agent after COVID-19. According to the World Health Organisation (WHO, 2025), an estimated 1.25 million deaths were attributed to TB in 2024 alone [1]. The disease is airborne and affects individuals of all ages and populations, while it is most prevalent among those with weakened immune systems, malnutrition, or tobacco dependence. It is estimated that nearly one-quarter of the global population carries latent M. tb infection, and approximately 5–10% of these individuals will develop active TB during their lifetime [1].

Multidrug-resistant tuberculosis (MDR-TB), characterised by resistance to isoniazid and rifampin, the two most effective first-line drugs, and extensively drug-resistant tuberculosis (XDR-TB), which exhibits additional resistance to fluoroquinolones and at least one second-line injectable drug, present a serious threat to global TB control efforts. In 2023, nearly 10% of reported cases in the United States were of MDR-TB [2]. This alarming trend underscores the urgent need for discovering novel anti-TB therapeutics with a new mechanism of action to overcome resistance.

Marine natural products have long served as invaluable sources of medicines, and new bioactive scaffolds. The deep-sea biosphere, particularly regions exceeding 200–6000 m in depth, represents one of the most chemically diverse and underexplored habitats on Earth [3]. Characterised by high pressure, low temperature, and limited oxygen and light, this gives rise to structurally distinct metabolites with exceptional pharmacological potential [4].

**Figure 1:**
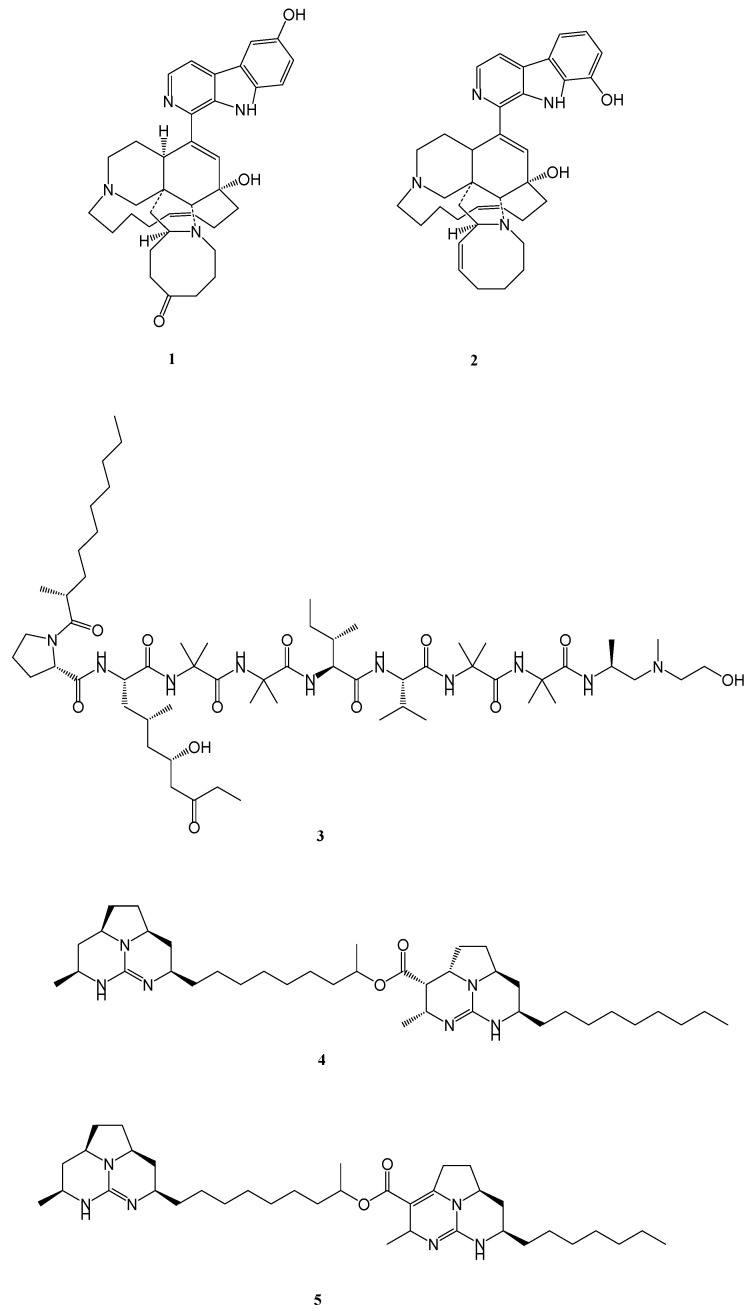
Structure of marine-derived metabolites with anti-mycobacterial **1**. 6-hydroxymanzamine E, **2**. 8-Hydroxymanzamine A, **3**. Trichoderin A, **4**. Batzelladine L, and **5**. Batzelladine N.

Several marine-derived natural products have been reported as antimicrobial, antitumor, and anthelminthic agents, with many progressing into preclinical and clinical evaluation, owing to their unique mode of action and chemical novelty [5,6,7]. Their activity spans a wide therapeutic spectrum from inhibiting pathogenic bacteria to suppressing cancer cell proliferation and parasite growth, showing the immense potential of the marine biosphere. An increasing number of marine metabolites and their derivatives have also been identified with anti-mycobacterial activity, supporting the exploration of marine metabolites as promising anti-tubercular candidates. For example, manzamine alkaloids, 6-hydroxymanzamine E and 8-hydroxymanzamine A, isolated from the marine sponge *Acanthostrongylophora* sp. [8,9,10], trichoderin A from marine-sponge-derived *Trichoderma* sp. [11], and batzelladine alkaloids L and N from sponge *M. unguifera* [12,13] (Figure 1) highlight the enormous potential of marine scaffolds in anti-tubercular drug discovery.

Despite this chemical diversity, the experimental exploration of marine natural products is limited by several practical challenges. Traditional bioassay-guided isolation requires substantial biomass, which is often expensive, yields low quantities of metabolites, and often results in the isolation of known metabolites. Also, many deep-sea or symbiotic microorganisms are uncultivable under standard laboratory conditions, and their biosynthetic potential remains largely inaccessible. Integrating metabolomics, genome mining, and computational chemistry can overcome these limitations by identifying and prioritising bioactive scaffolds prior to isolation or synthesis.

To bridge this translational gap, Computational-aided Drug Discovery (CADD) approaches have become indispensable tools for rapid prioritisation of promising leads. Structure-based virtual screening (SBVS), molecular docking, ADMET analysis, and molecular dynamics (MD) simulations enable the speedy evaluation of large chemical libraries. These methods predict binding affinities, selectivity, stability, and pharmacokinetic properties, narrowing thousands of candidates to the few promising ones for in vitro experimental validation.

Such in silico pharmacokinetic strategies have been successfully applied in antiviral and antifungal research. For example, screening 2033 marine natural products against SARS-CoV-2 replication enzymes (3CLpro, PLpro, and RdRp) identified two potent hit compounds with superior stability and pharmacokinetic properties compared to standard inhibitors [14]. Similarly, mining over 1400 marine natural products identified anti-EBV (Epstein–Barr Virus) candidates via docking and MD simulations. Recent computational investigations have also explored diverse therapeutic domains, from repositioning vincetene as a neuroprotective agent [15], designing novel 1,2-dihydroquinoline derivatives for multiple sclerosis [16], to developing indole-based scaffolds with antibacterial and antioxidant potential [17]. Extending these approaches to tuberculosis, virtual screening of the CMNPD library yielded selective inhibitors of InhA, a key enzyme in mycolic acid biosynthesis [18]. Furthermore, analyses of 139 marine fungal metabolites have shown that the majority (131) exhibit measurable anti-mycobacterial activity, highlighting the value of computational screening in guiding future TB drug discovery efforts [19]. Although these studies have not been validated in vitro, they provide a prominent base for rational hit prioritisation and further biological testing.

Building on these methodological advances, the present study employed an integrated computational strategy to discover novel anti-tubercular candidates from marine-derived compounds collected from depths exceeding 200 m. A curated library of 2773 deep-sea compounds sourced from the MarinLit, Reaxys, and CMNPD databases was systematically screened against a single validated M. tb target, Rv1155 [20]. Rv1155 is a pyridoxine-5′-phosphate oxidase-like (PNPOx) enzyme that binds the unique cofactor F_420,_ mediating redox reactions critical for M.tb survival [21].

Initial high-throughput molecular docking for preliminary binding assessment was followed by ADMET-based profiling to prioritise compounds with favourable drug-like properties, and by molecular dynamics (MD) simulations to evaluate the conformational stability and persistence of protein–ligand interactions under dynamic conditions.

This systematic multi-tier approach highlights the deep-sea marine environment as an underexplored yet promising reservoir of structurally diverse scaffolds with potential anti-tubercular activity. Beyond identifying promising candidates, the study establishes a cost-effective and generalised computational workflow for exploring marine metabolites diversity in the discovery of new inhibitors targeting the F_420_-dependent redox system of *Mycobacterium tuberculosis*, thereby contributing to the global effort to combat drug-resistant tuberculosis.

To the best of our knowledge, this study presents a novel attempt to systematically screen a comprehensive library of deep-sea-derived natural products against F_420_-dependent oxidoreductase Rv1155 of *M. tuberculosis.* Previous computational and medicinal studies have primarily focused on conventional drug targets such as InhA [22] or DprE1 [23], while the F_420_ enzyme system, although abundant and biologically significant, largely remains unexplored for anti-tubercular drug discovery [24]. This work addresses this gap by introducing novelty in both target selection and the application of a multi-tiered screening pipeline to explore marine chemical space, identifying F_420_-dependent enzyme inhibitors.

## 2. Results and Discussion

### 2.1. Molecular Docking Analysis

Molecular docking serves as a critical initial filter in structure-based drug discovery, providing rapid insight into protein–ligand relative binding affinities. It explores ligand conformations within the protein’s active site to identify the most favourable pose, ranked by a scoring function that estimates binding free energy [25]. However, limitations persist regarding their predictive accuracy for complex natural products. Scoring functions in molecular docking often oversimplify protein flexibility or solvent effects, leading to a disparity between theoretical and experimental affinities, especially in large molecules. Likewise, pharmacokinetic models are typically trained on smaller synthetic drug-like compounds, potentially limiting their precision when extrapolated to natural product scaffolds [26,27]. Nonetheless, the convergence of a multi-tier computational framework, combining molecular docking, ADMET profiling, MD simulations, and, more recently, AI-assisted scoring and binding free energy refinement, has greatly improved the reliability and cross-validation of virtual hits [28].

In this study, *Mycobacterium tuberculosis* (M. tb) enzyme Rv1155 (PDB ID: 4QVB) was selected as the sole molecular target for virtual screening owing to its confirmed role as a novel coenzyme F_420_-binding protein [21,29]. Rv1155 utilises cofactor F_420_ (denoted as F_4201_ and F_4202_ in the crystal structure) as an essential redox mediator, a system unique to actinobacteria and some archaea. The enzyme is involved in multiple M. tb metabolic pathways, including redox homeostasis, lipid metabolism, oxidative stress resistance, and activation of nitroimidazole prodrugs such as pretomanid and delamanid [21,30,31]. Because F_420_-dependent enzymes are absent in humans, this cofactor system offers a highly selective and mechanistically distinct therapeutic target for anti-tubercular drug design.

**Figure 2:**
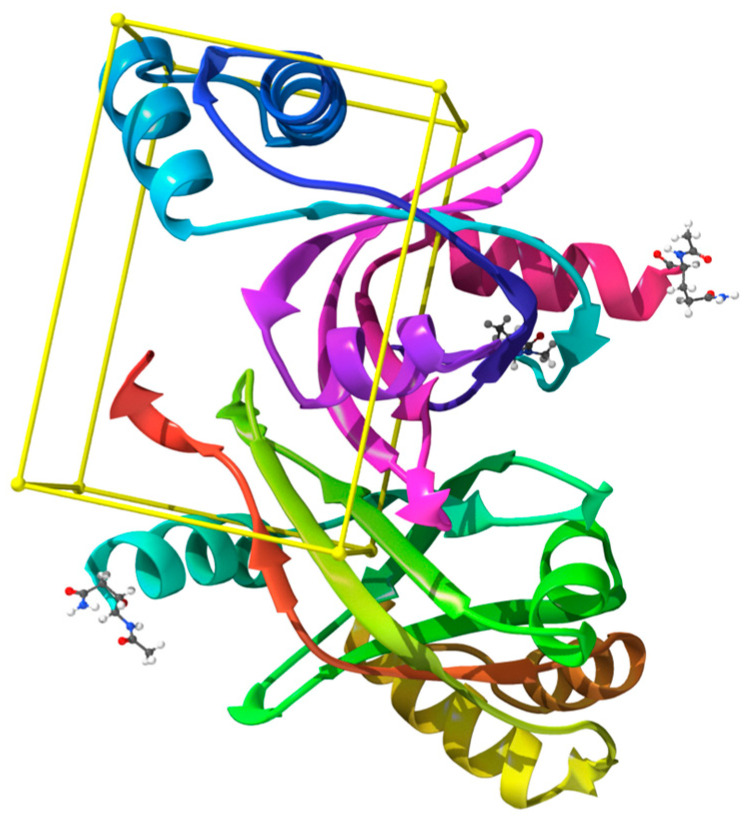
Crystal structure of *Mycobacterium tuberculosis* protein Rv1155 in complex with co-enzyme F_420_ (PDB ID: 4QVB); the yellow box shows the binding site (colour figure online). The grid box centre and dimensions were set to 10.8 × 20.1 × 18.6 Å and 31.4 × 21.8 × 24.4 Å.

The crystal structure of Rv1155 complexed with co-enzyme F_420_ (PDB: 4QVB, 1.8 Å resolution) reveals a Rossmann-like fold with a well-defined, hydrophobic binding cleft that accommodates the deazaflavin cofactor. This experimentally validated F_420_ binding pocket, identified from the co-crystallised F_4202_ ligand and confirmed using LigandScout pocket detection, was used to define the grid centre for docking (Figure 2). The grid dimensions were optimised to encompass the entire cofactor-binding cavity and adjacent residues at the dimer interface, ensuring sufficient sampling of larger, structurally diverse marine metabolites while avoiding irrelevant surface regions. Grid size adequacy was further supported by successful reproduction of the native F_4202_ binding pose during docking validation. Key residues shaping this site include Arg30, His27, Asn60, Arg55, Lys57, Trp77, and Tyr79 [21]. These residues define a rational pocket for ligand docking; therefore, targeting this cofactor-interacting region could disrupt essential redox reactions, ultimately compromising bacterial viability.

Initially, two cofactors, F_4201_ and F_4202_, were evaluated as docking standards; however, only F_4202_ achieved precise pocket alignment and RMSD validation and was therefore used as the reference ligand for further analysis. In our study, a total of 2773 deep-sea-derived compounds from the CMNPD, Reaxys, and MarinLit databases were docked against Rv1155, using the native cofactor (F_4202_) as a positional reference for grid definition with AutoDock Vina (v1.2.0). Although AutoDock Vina treats the receptor as rigid while allowing complete ligand flexibility, it neglects subtle conformational adjustments described by the induced-fit model. Yet, it remains highly effective as an initial screening tool for rapid identification of top hit candidates with favourable binding geometries [32,33]. For each compound, the lowest-energy conformation among nine binding poses was selected to represent the predictive complex.

Docking scores revealed binding affinities ranging from −0.59 to −15.9 kcal/mol, with several marine metabolites exhibiting comparable or superior scores to the native cofactor F_4202_ (−6.385 kcal/mol). Of the 2773 screened compounds, ligands from the CMNPD database and the combined MarinLit-Reaxys databases were analysed independently to ensure balanced representation from both curated sources. In total, 29 ligands displayed strong binding energies between −10.98 and −15.95 kcal/mol, exceeding the docking score of native cofactor F_4202_ (Table S1). These top-scoring compounds were subsequently advanced to ADMET profiling and molecular dynamics (MD) simulations to validate their pharmacokinetic profiles and dynamic stability further.

Visualisation of docking interactions revealed that several compounds occupied the same binding site as native cofactor F_4202_ in the Rv1155 protein. The residues MetA24, Tyr120, TyrA107, ValA36, and IleA51 formed a conserved hydrophobic pocket, interacting with several compounds through strong van der Waals forces. This mimics the hydrophobic anchoring observed in standard F_4202_. Notably, the residue SerA50 acts as a critical anchor, forming hydrogen bonds with multiple top-docked compounds, as seen in standard F_4202_. While the standard F_4202_ exhibits extensive electrostatic interactions with residues LysA57, ArgA129, and ArgA55 via its phosphate tail, the library compounds compensate for this lack of charge by maximising hydrophobic interactions. As summarised in Table 1, all three selected marine-derived ligands reproduced key cofactor-binding contacts with a distinct interaction profile. Upenamide (CMNPD _22964) exhibited the most balanced network involving both hydrogen bonding and extensive hydrophobic contacts that spanned the cofactor binding cleft. Aspyronol (Compound_1749) closely mimicked the native cofactor’s hydrogen bonding pattern which correlated with its high docking affinity and favourable binding orientation within the active site, while Fiscpropionate F (Compound_1796) favoured compact hydrophobic packing complemented by limited polar interactions.

**Figure 3:**
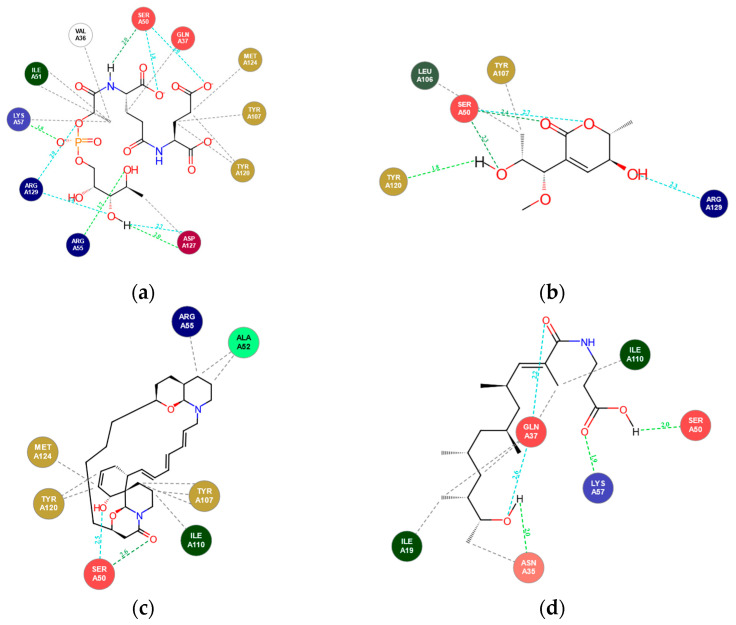
Two-dimensional interaction maps of (**a**) F_4202_ and selected top-docked compounds, (**b**) Compound_1749, (**c**) CMNPD_22964, and (**d**) Compound_1796 within the Rv1155 active site, showing hydrogen bonding (green/blue) and hydrophobic interactions (grey).

Overall, these results indicate that the identified marine metabolites effectively mimic essential F_4202_ interactions, particularly involving SerA50, ArgA55, and LysA57, while also introducing additional hydrophobic stabilisation that may enhance binding strength and conformational stability. Representative 2D interaction diagrams for the standard cofactor and selected top-docked ligands are shown in Figure 3 and Table 1 describes key hydrogen bond and hydrophobic residues of each ligand during interaction.

#### Validation of Docking Methodology

**Figure 4:**
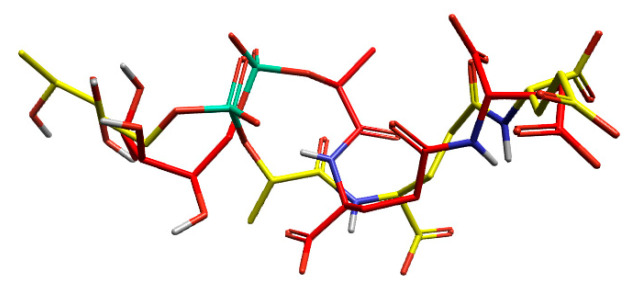
The comparison between the experimental and reference poses of the co-crystallised ligands validates the docking method showing docked ligand pose in yellow and ligand pose extracted from crystal structure in red.

The docking method was validated by docking the co-crystallised ligand into the protein’s active site and superimposing the docked conformer with the co-crystallised conformer (Figure 4). The low RMSD of 2.77 Å between the experimental and co-crystallised ligand indicated the same orientation, validating the docking method.

### 2.2. ADMET Evaluation

A total of 29 compounds with the highest docking affinities were prioritised for ADMET evaluation to ensure that the computationally identified hits are also chemically and biologically viable for further optimisation and experimental validation.

ADMET predictions were performed based on Lipinski’s rule of five, the Pfizer rule, and the Predicted Blood–Brain Barrier permeability (PBB) parameter. Lipinski’s rule was applied to assess oral bioavailability, while the Pfizer rule served as an early toxicity filter. The PBB parameter was specifically incorporated owing to its pharmacological relevance in tuberculosis, where effective CNS penetration must be balanced with the risk of neurotoxicity.

Although pulmonary infection is the primary manifestation of TB, *M. tuberculosis* can disseminate to extrapulmonary sites, including the central nervous system (CNS), causing tuberculous meningitis (TBM). This form remains one of the most challenging to treat due to the limited blood–brain barrier (BBB) permeability of most frontline drugs, such as ethambutol and streptomycin, which results in subtherapeutic CNS concentrations and poor clinical outcomes [34,35]. Therefore, incorporating the PBB filter into ADMET analysis enables early identification of compounds with an optimal physicochemical balance, capable of sufficient intracellular penetration without excessive BBB permeation, thereby minimising CNS-related adverse effects.

**Figure 5:**
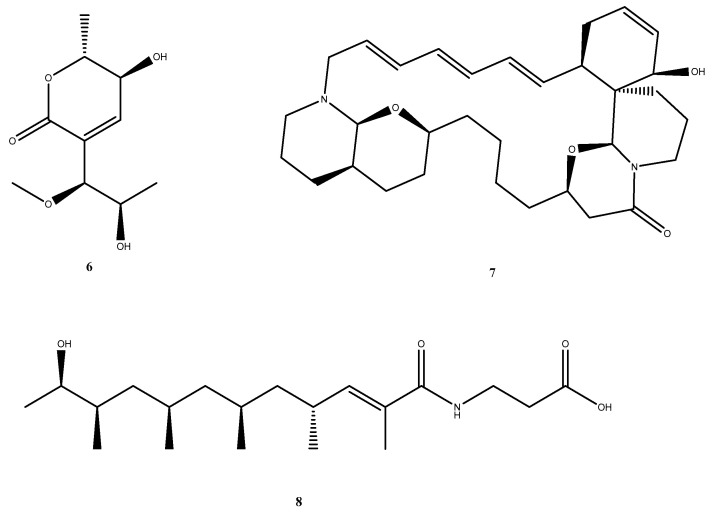
Structure of three lead compounds selected for molecular dynamics simulation: **6.** Aspyronol/Compound_1749 (PubChem CID: 139585929), **7**. Upenamide/CMNPD _22964 (PubChem CID: 10792140), and **8**. Fiscpropionate F/Compound_1796 (PubChem CID: 155524686).

Of the 29 compounds evaluated, most satisfied both Lipinski and Pfizer rules, indicating favourable oral bioavailability and safety profiles (Table S2). The remaining seven violated the Pfizer rule and were excluded from further analysis. Among the 22 compounds with acceptable drug likeness, three ligands were further prioritised based on their PBB values: Upenamide (CMNPD_22964; PubChem CID: 10792140) from the CMNPD dataset, Aspyronol (Compound_1749; PubChem CID: 139585929), and Fiscpropionate F (Compound_1796; PubChem CID: 155524686) from the combined MarinLit-Reaxys dataset (Figure 5).

Among these, Aspyronol displayed a PBB value (~21) remarkably close to that of the native cofactor F_4202_ (~29). Structural comparison revealed that Aspyronol possesses a highly oxygenated, polyhydroxylated scaffold with ether linkages and chiral centres at secondary alcohol positions. These polar functional groups mimic the hydrogen bonding pattern and polarity distribution of F_4202_, causing similarly low membrane permeability. Both molecules exhibit low lipophilicity and extensive hydrogen bond donor/acceptor networks, which restrict passive BBB diffusion while maintaining strong cytosolic retention. This structural and physicochemical balance suggests that Aspyronol may mimic the intracellular distribution profile of F_4202_, enabling effective targeting of Rv1155 while limiting neurotoxicity.

It is noteworthy that the native cofactor F_4202_ does not comply with Lipinski’s rule of five, primarily due to its high molecular weight, multiple phosphate and hydroxyl groups, and large polar surface area. However, this deviation does not diminish its biological importance, as F_4202_ is an endogenously synthesised cofactor that functions through specific binding rather than passive diffusion across membranes. While Lipinski’s criteria are essential for small-molecule drug discovery, the physicochemical characteristics of F_4202_ define the chemical environment and polarity preferences of the Rv1155 binding site.

The ADMET analysis here underscores that effective Rv1155 inhibitors should balance polarity and lipophilicity to achieve selective intracellular accumulation without excessive BBB penetration. Aspyronol, in particular, demonstrates the desirable combination of cofactor- like polarity and favourable pharmacokinetics. Overall, the ADMET evaluation showed that Upenamide, Aspyronol, and Fiscpropionate F displayed a promising pharmacokinetic profile and balanced permeability, supporting their progression to molecular dynamics simulations for further validation of stability and binding persistence.

### 2.3. Molecular Dynamics (MD) Simulation

We performed 200 ns molecular dynamics simulations on the apoprotein (PDB ID: 4QVB) and its complexes with Aspyronol, Fiscpropionate F, Upenamide, and Standard F_4202_ compound using Flare v9.0. The stability of each system was assessed through Root Mean Square Deviation (RMSD), Root Mean Square Fluctuation (RMSF), radius of gyration (Rg), and principal component analysis (PCA). RMSD is a measure of the system’s movement and overall stability, while RMSF is used to measure molecular flexibility. The radius of gyration is used to assess the system’s compactness and to track changes in the shape of the molecule. At the same time, PCA identifies the key collective motions in the simulation.

#### 2.3.1. Root Mean Square Deviation (RMSD)

**Figure 6:**
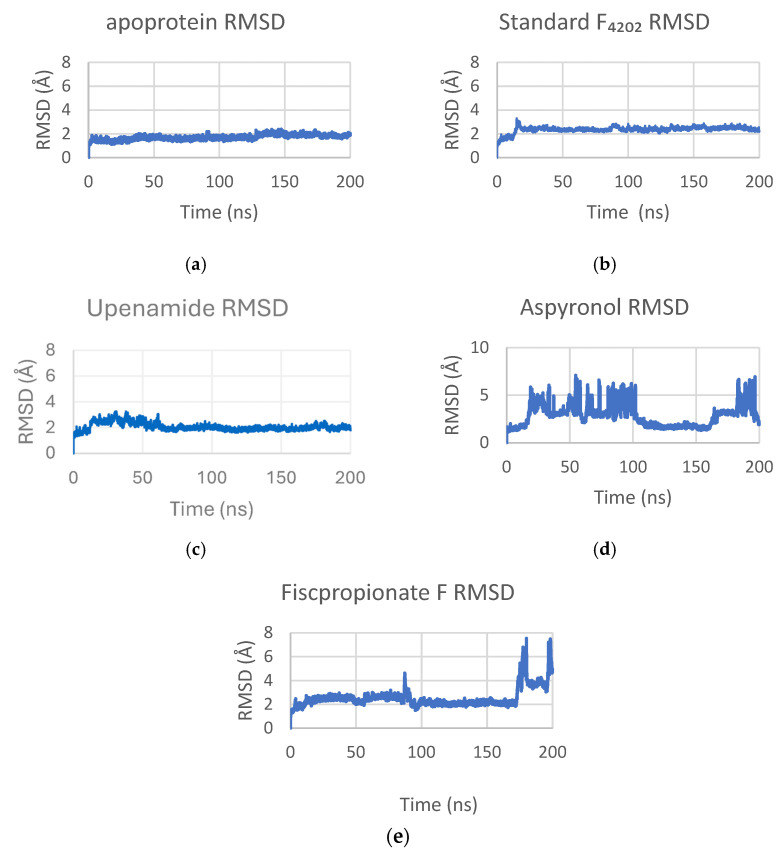
RMSD profiles of (**a**) apoprotein, (**b**) standard F_4202_, (**c**) Upenamide, (**d**) Aspyronol, and (**e**) Fiscpropionate F during simulation. It should be noted that the Aspyronol complex shows significant deviation throughout the simulation.

The apoprotein demonstrated a stable conformation, with a low mean RMSD of 1.70 ± 0.21 Å. Complexes with F_4202_ and Upenamide also exhibited stable trajectories, with RMSD values of 2.36 ± 0.25 Å and 2.06 ± 0.30 Å, respectively. In contrast, Aspyronol complex (RMSD 2.90 ± 1.20 Å) displayed significant transient deviations between 20–100 ns and 180–200 ns, suggesting a potential conformational shift as shown in Figure 6. Similarly, the Fiscpropionate F complex (RMSD 2.54 ± 0.81 Å) was largely stable but showed a substantial deviation near the simulation’s end, indicating late-onset structural instability or flexibility. In addition, Fiscpropionate F complex shows a brief deviation between 85 and 95 ns, suggesting a transitory conformation explored during that time.

The RMSD profiles collectively suggest that Upenamide forms the most conformationally stable complex with Rv1155, maintaining structural alignment similar to the native cofactor. In contrast, the transient deviation observed for Aspyronol and Fiscpropionate F implies partial flexibility within the binding pocket, which could be fine-tuned through side chain modification or ring constraint to enhance structural persistence. The stability of the Upenamide-Rv1155 complex supports its candidacy as lead scaffold for designing F_420_-mimicking inhibitors with high conformational fidelity.

#### 2.3.2. Root Mean Square Fluctuation (RMSF)

**Figure 7:**
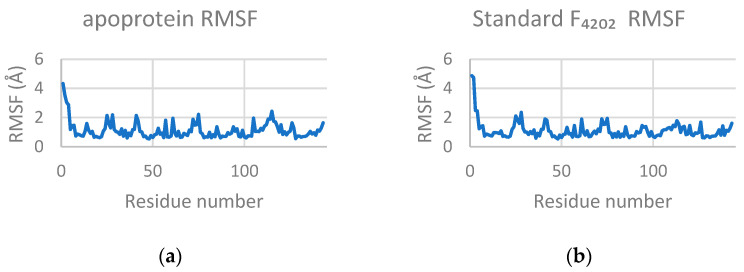

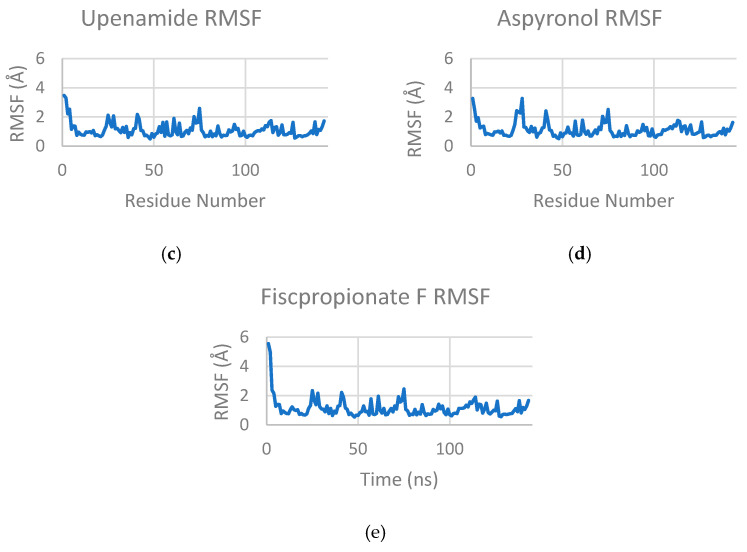
The RMSF profiles of (**a**) apoprotein (1.11 ± 0.57 Å), (**b**) standard F_4202_ (1.10 ± 0.59 Å), (**c**) Upenamide (1.09 ± 0.49 Å), (**d**) Aspyronol (1.11 ± 0.50 Å), and (**e**) Fiscpropionate F (1.13 ± 0.64 Å) of each of the trajectories. Each profile shows that Residues 1 and 2 have significantly higher values than the others.

The RMSF was comparable across all systems, with the apoprotein and its complexes with F_4202_, Upenamide, Aspyronol, and Fiscpropionate F exhibiting values very close to each other. In each simulation, residues 1 and 2 displayed elevated flexibility, suggesting their contribution to the overall protein dynamics (Figure 7).

**Figure 8:**
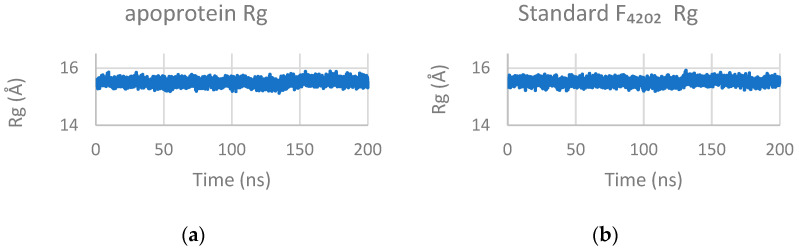

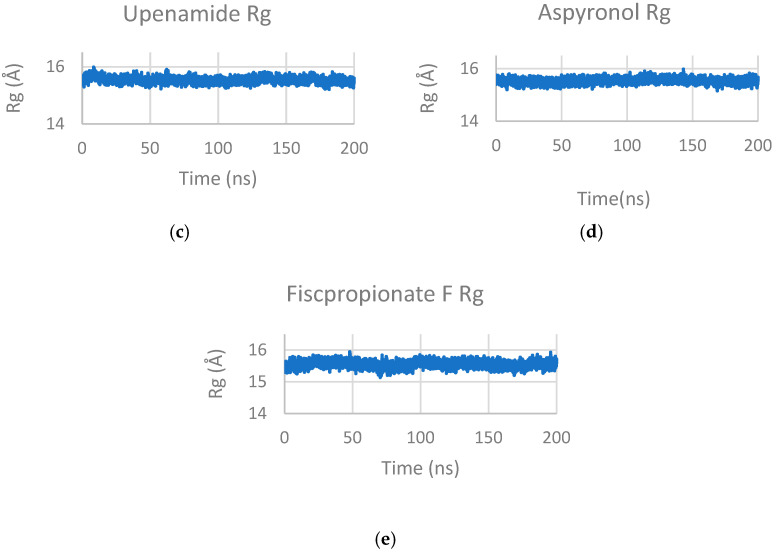
The radius of gyration (R_g_) profiles for each of the trajectories of (**a**) apoprotein, (**b**) standard F_4202_, (**c**) Upenamide, (**d**) Aspyronol, and (**e**) Fiscpropionate F. They are observed to maintain a steady value over time in simulations.

The radius of gyration (Rg) remained stable throughout 200 ns simulation, averaging 15.50 ± 0.10 Å across all systems (Figure 8), confirming overall compactness of protein structure upon ligand binding.

The uniform RMSF and Rg patterns indicate that ligand association does not compromise Rv1155’s structural integrity. The limited fluctuations confined to the N-terminal residues are unlikely to affect catalytic function, supporting that all three selected compounds bind within a structurally rigid region of the protein while achieving strong binding affinity. This underscores the compounds’ potential as a stable redox enzyme inhibitor.

#### 2.3.3. Principal Component Analysis (PCA)

Principal component analysis (PCA) of the trajectories revealed the systems’ large-scale collective motions. The apoprotein, and F_4202_ complexes, exhibited predominantly restricted conformational sampling, indicating stable dynamics with minor transient deviations observed between 130 and 150 ns, and 170 and 190 ns, respectively. Similarly, the Upenamide complex is observed to be strictly restricted to a smaller conformational space and fewer deviations consistent with its low RMSD fluctuations.

**Figure 9:**
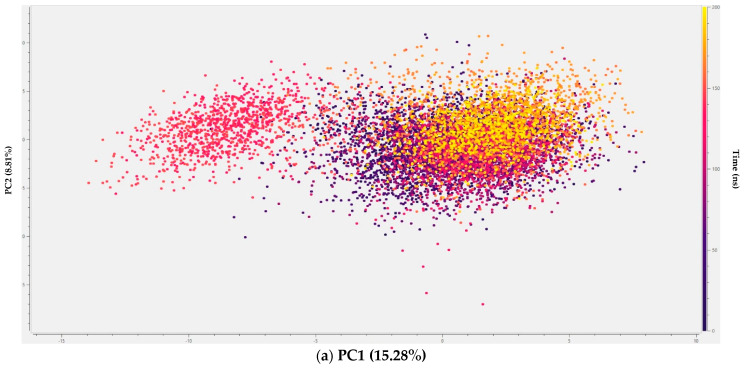

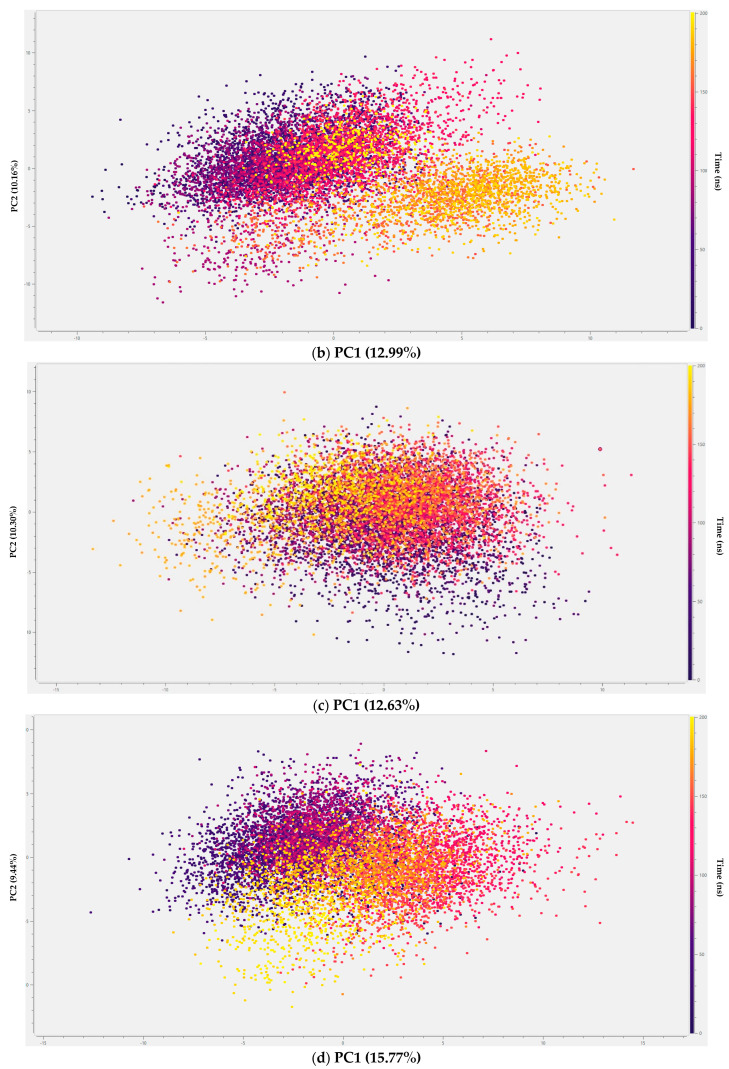

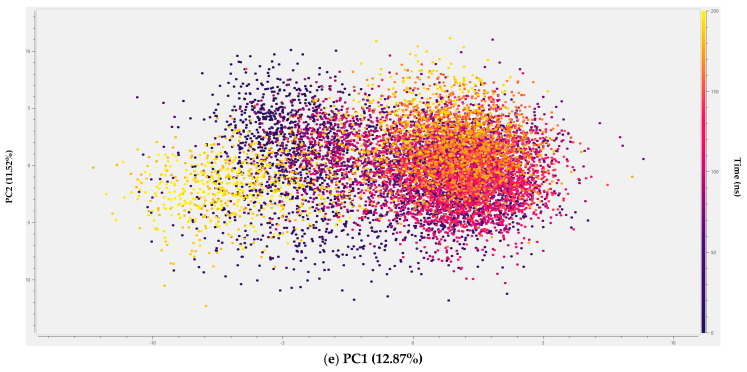
PCA analyses of (**a**) apoprotein, (**b**) standard F_4202_, (**c**) Upenamide, (**d**) Aspyronol, and (**e**) Fiscpropionate F. Unlike its RMSD profile, the Aspyronol complex shows slight variation, suggesting that the protein oscillates between two stable conformations.

In contrast, the Aspyronol complex, though similarly restricted, displayed oscillatory motion and local flexibility, while the Fiscpropionate F complex exhibits a major conformational shift within the reduced PCA space, transitioning to and stabilising in a distinctly different region by the end of the simulation. All PCA plots are presented in Figure 9. 

Overall, PCA results show that Upenamide and F_4202_ maintain the most restricted conformational space, reflecting the stable binding and minimal induced fit. Aspyronol shows limited flexibility compatible with reversible binding, while Fiscpropionate F exhibits higher flexibility, suggesting weaker site retention. For inhibitor design, ligands that constrain Rv1155 dynamics like Upenamide are likely to confer superior binding stability and specificity by minimising large-scale protein motions.

### 2.4. Hydrogen Bond Contacts

**Figure 10:**
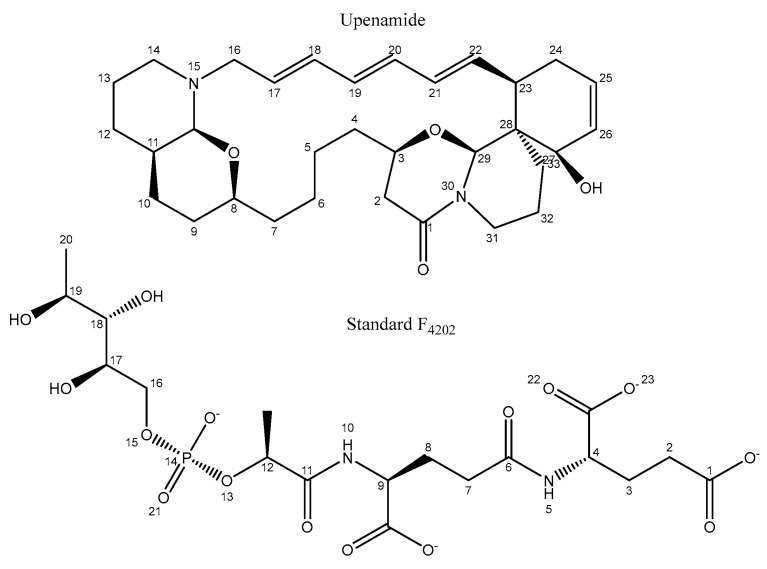
The structure of Upenamide and standard with positions annotated according to the standard numbering system.

The hydrogen bond contacts were also calculated for the four complexes, with only the Upenamide and the Standard F_4202_ complexes showing significant (>50% of the frames) contacts with residues in the protein. In the Upenamide, the ketonic oxygen at position 1 (please refer to Figure 10 for the structure and numbering) showed significant contact with two protons. One of the contacts observed was with the hydroxyl proton on the aromatic ring of the TyrA120 residue for 65.6% of the frames, and the other was with the hydroxyl proton on the backbone of the SerA50 residue for 67% of the frames (Table 2). In both cases, the frames with hydrogen bond contacts were concentrated beyond the 60 ns time point.

Similarly, in the F_4202_-Rv1155 complex, oxygen at position 21 forms a hydrogen bond contact with the terminal amide carbon of the AsnA35 residue for 50.0% of the frames while oxygens at positions 22 and 23 interacted with protons in the ArgA129 residue, as summarised in Table 3. A hydrogen bond persistence map was generated to visualise temporal stability of key interactions throughout the 200 ns simulation. Darker or continuous bands on maps indicate stable hydrogen bonds maintained for larger fraction of trajectory, whereas lighter or intermittent patterns represent transient contacts that form and break during the simulation.

The persistence of the hydrogen bond with SerA50 and TyrA120 in the Upenamide complex mirrors the anchoring interactions of the native cofactor, suggesting that these residues are critical for maintaining catalytic-site stability. This strong and sustained hydrogen bond network likely supports the conformational stability observed in MD simulation, highlighting Upenamide as a structurally compatible mimic of F_4202_ and promising lead scaffold for optimising redox-enzyme inhibitors.

### 2.5. MM-GBSA Analysis

MM-GBSA calculations were conducted to estimate the binding free energy of the top-docked Rv1155 ligand complexes. The results summarised in Table 4 revealed that all three candidates exhibited markedly stronger binding affinities ranging from −24.77 to −34.07 kcal/mol. Among these, Fiscpropionate F showed the most favourable Δ*G* (−34.07 kcal/mol), followed by Upenamide and Aspyronol. The non-polar solvation energies (ranging from −30.45 to −40.06 kcal/mol/Å^2^) and corresponding SASA values (6262–8184 Å^2^) indicated extensive hydrophobic interactions stabilising the complexes.

The favourable MM-GBSA scores across all three ligands highlight the importance of hydrophobic packing and van der Waals interaction in stabilising inhibitors within the Rv1155 active site. Although Fiscpropionate F showed highest affinity, its conformational variability in MD simulations suggests an interplay between binding strength and structural persistence. Conversely, Upenamide, with slightly weaker ΔG but greater dynamic stability and persistent hydrogen bonding, represents a more balanced and pharmaceutically viable scaffold for further optimisation.

The native cofactor F_4202_ was excluded from the MM-GBSA comparison because its multiple phosphate groups confer a highly charged, structurally distinct framework, rendering direct energetic comparisons with small-molecule ligands unreliable. This ensures that the calculated binding energies reflect realistic and internally consistent trends among the marine-derived metabolites.

### 2.6. Proposed Molecular Mechanisms of Inhibition

Based on docking, interaction profiling, and molecular dynamics analyses, a putative inhibition mechanism is proposed for the identified deep-sea metabolites against Rv1155. This enzyme, an F_420_-dependent oxidoreductase, relies on the productive binding of the F_420_ cofactor within its conserved pocket for redox catalysis. Since virtual screening was performed using the cofactor-bound Rv1155 structure (PDB: 4QVB), the selected metabolites were prioritised for their ability to occupy the same pocket as F_4202_.

Docking analyses revealed that the top compounds interact with key cofactor-recognition residues, including SerA50, TyrA120, and surrounding hydrophobic residues that stabilise the deazaflavin core of F_420_. Unlike the native cofactor, which forms extensive electrostatic interactions through its phosphate tail, the identified metabolites compensate via strong van der Waals contacts and hydrogen bonding within the binding cleft.

Molecular dynamics simulations further support this model: Upenamide remained stably anchored within the pocket over 200 ns, forming persistent hydrogen bonds with SerA50 and TyrA120, consistent with a sustained inhibitory binding mode. In contrast, Aspyronol and Fiscpropionate F display more transient polar contacts while remaining pocket-associated, suggesting alternative but still competitive binding modes driven largely by hydrophobic interactions.

Together, these findings suggest a competitive inhibition mechanism in which the metabolites occupy the F_420_-binding pocket, preventing cofactor association and thereby impairing Rv1155′s redox activity. While experimental validation is required to confirm, the convergence of computational data provides a coherent molecular rationale for this inhibitory mode of action.

Interestingly, all three lead compounds identified in this study are naturally occurring marine metabolites with previously reported antimicrobial activity. Upenamide (CMNPD_22964) was first isolated from the sponge *Echinochalina* sp., known for its antibacterial and cytotoxic potential [36]. Aspyronol (Compound_1749), a polyketide isolated from deep-sea *Aspergillus* sp., has shown cytotoxic, antibacterial, and antifungal activity [37]. Fiscpropionate F (Compound_1796), along with its analogue series, was reported from deep-sea Aspergillus fischeri FS452 and exhibited strong anti-mycobacterial activity. A series of these analogues demonstrated inhibition of M. tb protein tyrosine phosphatase B (MptpB), suggesting a reliable anti-tubercular activity [38]. In our study, Fiscpropionate F ranked among the top-docked compounds, passed ADMET filters, and underwent detailed MD and MM-GBSA analyses. This strong binding affinity observed towards the F_420_-binding oxidoreductase Rv1155 aligns with its reported anti-mycobacterial potential and supports the rationale for repurposing this scaffold for redox-based targeting of M. tuberculosis. Overall, these findings strengthen the ground for exploring deep-sea metabolites as novel anti-tubercular leads and provide a basis for experimental follow-up and future structure–activity optimisation.

### 2.7. Limitations of the Study

Although this work establishes a strong computational framework for identifying potential inhibitors of F_420-_dependent Rv1155, it remains limited by inherent constraints of in silico methodologies. The docking and MD simulations were performed on a single enzyme target, and therefore do not account for the full spectrum of F_420-_binding enzymes encoded by *M.tuberculosis*. Additonally the predictive accuracy of ADMET and MM-GBSA models may vary due to their dependence on training data derived primarily from synthetic compounds rather than complex natural product. Also experimental validation through enzyme inhibiton assays and in vitro anti-mycobacterial screening is essential to confirm the predictive activities and pharmacokinetic properties of hit compounds.

## 3. Materials and Methods

### 3.1. Source of Compounds

A total of 2773 deep-sea-derived natural compounds, isolated from depths exceeding 200 m, were screened against *Mycobacterium tuberculosis* (M. tb). The compound library [20] (in press) was curated from three comprehensive databases: MarinLit [39], Reaxys [40], and the Comprehensive Marine Natural Products Database (CMNPD) [41]. These sources collectively comprise structurally diverse marine metabolites with reported or predicted biological activity, providing a representative library for virtual screening.

### 3.2. Molecular Docking

All 2773 compounds were prepared as ligands using AutoDock Vina v1.2.0 (The Scripps Research Institute, La Jolla, CA, USA) [42,43] through Samson Connect (OneAngstrom, 2022) (https://www.samson-connect.net/) (accessed on 30 May 2025). Ligand geometry optimisation ensured that each molecule adopted a low-energy conformation suitable for reliable target interaction. The three-dimensional structure of *M. tuberculosis* Rv1155 enzyme in complex with its cofactor F_420_ (PDB ID: 4QVB) was retrieved from the RCSB Protein Data Bank (https://www.rcsb.org/) (accessed on 05 May 2025). Protein preparation involved removing water molecules and retaining a single cofactor conformation (F_4202_) for reference in grid generation. The receptor’s potential binding pocket was identified using LigandScout 4.4.8 [44]. The LigandScout pocket-finder algorithm detects a potential binding pocket by generating a three-dimensional grid over the protein surface and assigning a buriedness score to each grid point. Clusters of highly buried grid points are connected and enclosed by an isosurface, representing the spatial volume of a potential ligand-binding site. For Rv1155 (PDB ID: 4QVB), the grid box centre and dimensions were set to 10.8 × 20.1 × 18.6 Å and 31.4 × 21.8 × 24.4 Å, respectively.

Docking simulations were performed using AutoDock Vina v1.2.0. The binding mode number was set to 9, generating nine distinct poses for each ligand to increase the likelihood of capturing a biologically relevant orientation. The maximum energy difference between the best and other poses was limited to 3 kcal/mol. The exhaustiveness parameter was set to 4, and convergence was validated by repeating docking runs at exhaustiveness levels of 8 and 16, with identical parameters. The co-crystallised cofactor F_4202_ served as a positive control to validate docking accuracy by reproducing its experimental binding pose. Compounds obtained from the CMNPD dataset were screened separately from those obtained from the combined MarinLit-Reaxys dataset. The top-scoring ligands were then pooled and prioritised for further evaluation.

### 3.3. ADMET Study

Pharmacokinetic profiling of the top 29 docked deep-sea compounds was performed using ADMETlab 3.0 [45] web server. The SMILES notations of selected compounds were submitted to the platform to predict their absorption, distribution, metabolism, excretion, and toxicity (ADMET) properties. Drug-likeness assessment was based on three primary parameters: Lipinski’s rule of five (for oral bioavailability), Pfizer’s rule (for toxicity filtering), and Predicted Blood–Brain Barrier permeability (PBB) (for CNS penetration potential). The evaluation ensured that the selected compounds not only exhibited strong binding affinity but were also pharmacologically acceptable and had biologically viable profiles for further optimisation.

### 3.4. Molecular Dynamics (MD) Simulations

Molecular dynamics (MD) simulations were performed using Flare v9.0 (Cresset, Cambridge, UK) integrated with the OpenMM 7.7 package [46]. Simulations were conducted for 200 ns employing the Open Force Field with explicit TIP3P water molecules to replicate near-physiological conditions. Each protein–ligand complex was solvated in a truncated octahedral box with a 10 Å buffer distance from the protein surface.

Partial atomic charges for ligands were assigned using the AM1-BCC method, and energy minimisation was performed until an energy tolerance of 0.25 kcal/mol was reached to remove steric clashes. Equilibration was carried out for 200 ps, followed by production runs in the isothermal-isobaric ensemble at 298 K and 1 bar, using a four-fs integration time step. Trajectory analyses included Root Mean Square Deviation (RMSD) to assess complex stability, Root Mean Square Fluctuation (RMSF) to assess residue flexibility, and hydrogen bond occupancy to quantify persistent intermolecular interactions within the Rv1155 active site. Each simulation was repeated independently under identical conditions to confirm the reproducibility and consistency of the results.

### 3.5. MM-GBSA Calculations

The Molecular Mechanics–Generalised Born Surface Area (MM-GBSA) was used to estimate the binding free energy (∆*Gbind*) of top-ranked protein–ligand complexes following molecular dynamics (MD) simulations. All calculations were performed in Flare v9.0 (Cresset, Cambridge, UK) using OpenMM engine with Open Force Field (v2.2.0) [47]. The AM1-BCC method was used to calculate partial atomic charges for ligands, and the implicit solvation model GBn2 was used to account for solvent polarisation effects. Calculations were performed under normal calculation mode, and both protein and ligand were energy minimised before scoring.

The standard MM-GBSA formula calculated the binding free energy for each complex:

(1) ∆Gbind=Gcomplex−(Gprotein+Gligand)

where G includes molecular mechanistic energy, polar solvation energy, and non-polar solvation energy.

The non-polar contribution (Gsa) was calculated from SASA (Solvent-Accessible Surface Area) using a linear relationship [48]:

(2) Gsa=γ×SASA+β

(3) $$SASA=\left(Gsa-\beta \right)/\gamma$$

where β and γ are empirical constants set to 0.86 kcal/mol and 0.005 kcal/mol/Å^2^, respectively. MM-GBSA and SASA were calculated to assess the stability and thermodynamic favourability of binding within the Rv1155 active site. The calculations were performed on the lowest energy pose obtained from docking.

## 4. Conclusions

In this study, we explored the deep-sea metabolites as promising scaffolds for developing new anti-tubercular agents targeting the F_420_-dependent oxidoreductase Rv1155 of *Mycobacterium tuberculosis*. Screening of a total 2773 marine-derived secondary metabolites from the CMNPD, Reaxys, and MarinLit databases identified three hit candidates: Upenamide, Aspyronol, and Fiscpropionate F. Among these, Upenamide demonstrated most favourable binding affinity and stable protein–ligand interaction, while Aspyronol and Fiscpropionate F exhibited higher flexibility, indicating opportunities for optimisation. Aspyronol also displayed highly desirable ADMET profile, particularly for its low predicted BBB permeability relative to the native F_4202_ cofactor, suggesting minimal neurotoxicity. Notably, while this study focused on a single F_420_-dependent Rv1155 as a proof-of-concept, *M. tuberculosis* encodes at least 28 distinct F_420_-binding enzymes, each contributing to its redox potential and drug susceptibility [24]. Extending this approach to other F_420_-dependent enzymes could broaden understanding of redox-driven metabolic networks and accelerate the discovery of broad-spectrum, mechanism-based anti-TB therapeutics. Overall, these findings highlight the deep-sea metabolites as a promising source of anti-tubercular scaffolds and present a cost-effective approach for identifying F_420_-targeting inhibitors. This work provides a foundation for future in vitro and in vivo validation of these identified hit compounds. 

In alignment with the objectives of the UN Biodiversity Beyond National Jurisdiction (BBNJ) Agreement which entered into force on 17 January 2026, this study underscores the value of deep-sea biodiversity as a source of novel pharmaceutical leads. By linking molecular discovery to sustainable ocean governance, our findings contribute to the emerging framework for equitable exploration and benefit-sharing of marine genetic resources.

## Figures and Tables

**Table 1:** Ligands with key hydrogen bond residues and hydrophobic residues.

Ligand/Compound	Key Hydrogen Bonding Residues	Key Hydrophobic Interactions
Standard_F_4202_	SerA50, ArgA55, LysA57, ArgA129	TyrA107, TyrA120, MetA124, LeuA106
Aspyronol (Compound_1749)	SerA50, TyrA107, TyrA120, ArgA129	LeuA106
Upenamide (CMNPD _22964)	SerA50, ArgA55, AlaA52	MetA124, TyrA107, TyrA120, IleA110
Fiscpropionate F (Compound_1796)	SerA50, GlnA37, AsnA35, LysA57	IleA19, IleA110

**Table 2:** Hydrogen bond contacts along with their time series profile for the oxygen at Position 1 for Upenamide (each vertical line in the time series profile refers to a frame where the hydrogen bond contact was detected).

Residue Proton	Proportion of Frames (%)	Time Series Profile (0–200 ns)
Aromatic hydroxyl of TyrA120	65.6	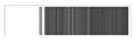
Backbone hydroxyl of SerA50	67.0	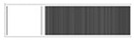

**Table 3:** Hydrogen bond contact of oxygen molecules of the standard complex (each vertical line in the time series profile refers to a frame where the hydrogen bond contact was detected).

Atom Number in Standard	Proton in Arg129(A)	Proportion of Frames (%)	Time Series Profile (0–200 ns)
O22	Imine guanidine proton	58.1	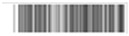
O23	Imine guanidine proton	54.8	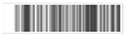
O22	Terminal guanidine proton	56.4	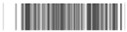
O23	Terminal guanidine proton	54.2	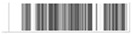

**Table 4:** MM-GBSA and SASA values for each complex.

Protein Complexed with	(kcal/mol)	Non-Polar Solvation Energy of (kcal/mol/Å^2^)	(Å^2^)
Aspyronol (Compound_1749)	−24.77	−30.45	−6262
Upenamide (CMNPD _22964)	−28.56	−40.06	−8184
Fiscpropionate F (Compound_1796)	−34.07	−30.50	−6272

## Data Availability

The data that supports the findings of this study are available from the corresponding author, M.J., upon reasonable request.

## Supplementary Materials

The following supporting information can be downloaded at: https://www.mdpi.com/article/10.3390/md24020058/s1, Table S1: The molecular docking scores of top-docked deep-sea compounds and standard against *Mycobacterium tuberculosis* (M. tb) protein Rv1155; Table S2: Predicted ADMET parameters of the top scored deep-sea compounds and standard using ADMET 3.0.

## Abbreviation

The following abbreviation is used in this manuscript:

TLA	Three-letter acronym
