# Supplementary material for: Deep-Sea Marine Metabolites as Promising Anti-Tubercular Agents: CADD-Guided Targeting of the F420-Dependent Oxidoreductase
Source: Mar Drugs. 2026 Jan 31;24(2):58. doi: 10.3390/md24020058 (PMC12942459; doi:10.3390/md24020058)
Supplement: Supplementary file 1 [file marinedrugs-24-00058-s001.zip › marinedrugs-4037425-Supplementary.pdf]

Table S1. The molecular docking scores of top docked deep-sea compounds and standard against *Mycobacterium tuberculosis* (Mtb) protein Rv1155

Compound ID	Structure	Docking score (kcal/mol)
**Compound_1749**	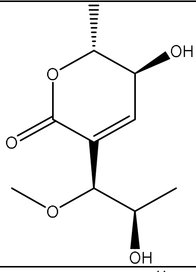	**-15.949**
Compound_1665	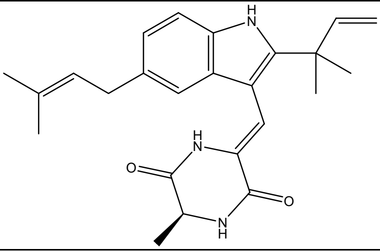	-14.554
Compound_1808	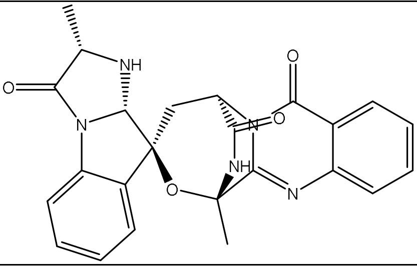	-13.76
Compound_1794	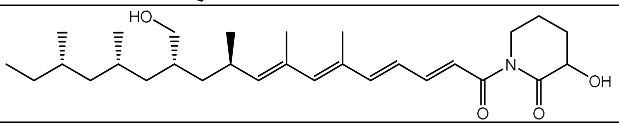	-12.422
Compound_1809	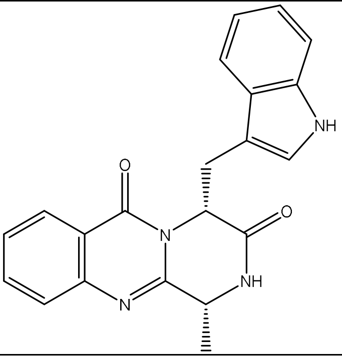	-13.285
Compound_1540	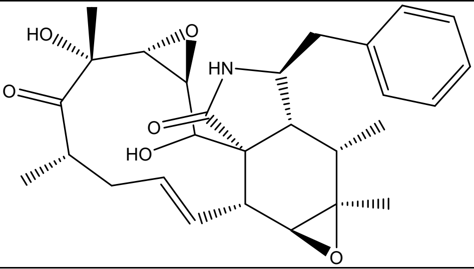	-13.225

Compound_1786	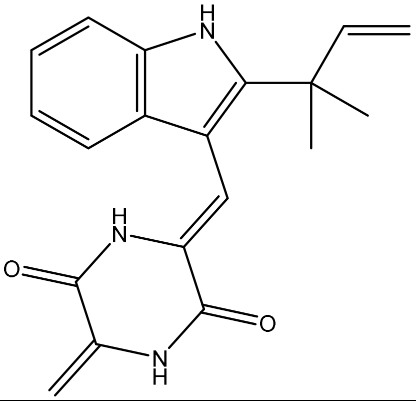	-14.035
**Compound_1796**	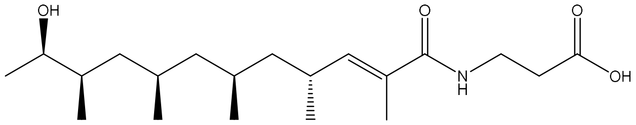	**-14.402**
Compound_1552	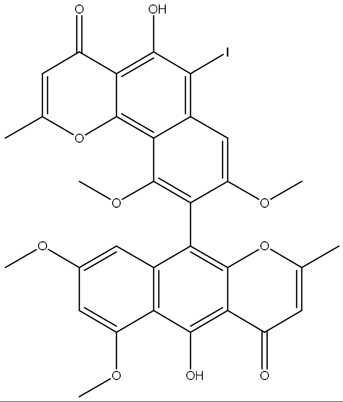	-12.82
Compound_1799	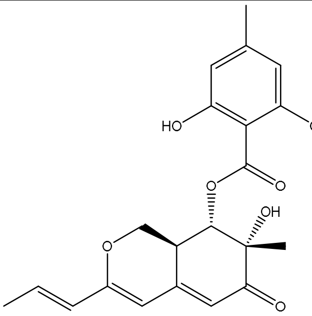	-13.022
Compound_1539	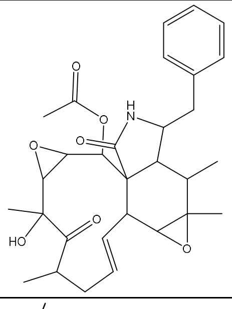	-12.538
Compound_1529	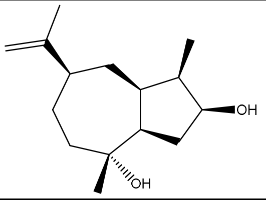	-12.495

Compound_1745	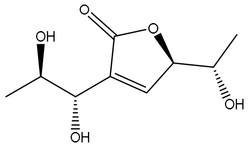	-11.127
Compound_1639	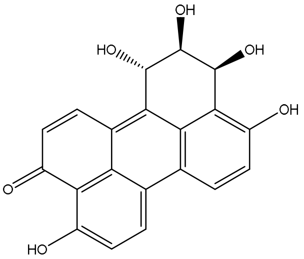	-12.477
Compound_1757	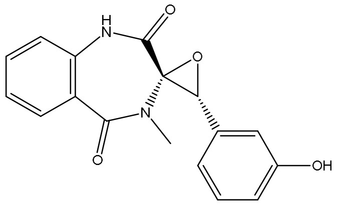	-12.454
Compound_1744	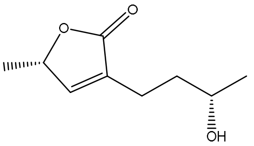	-12.415
Compound_1624	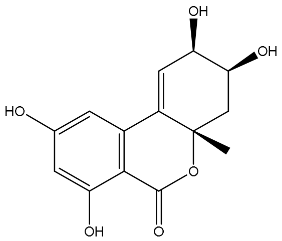	-12.383
Compound_1522	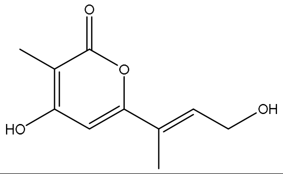	-12.546
Compound_1816	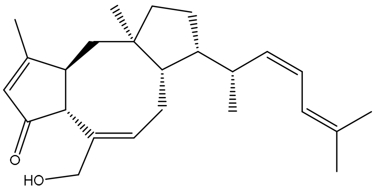	-12.195
Compound_1655	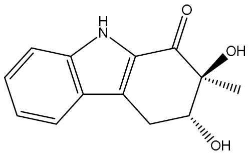	-12.11
Compound_1763	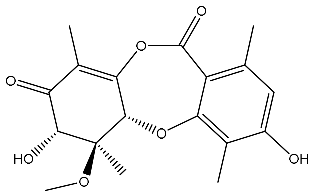	-12.114

Compound_1654	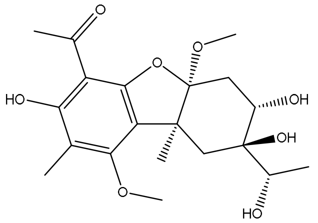	-12.118
Compound_1802	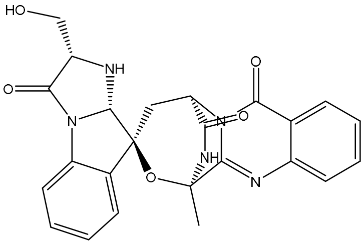	-12.861
CMNPD _14553	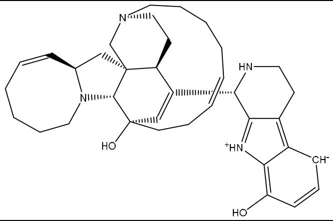	-11.544
CMNPD _16909	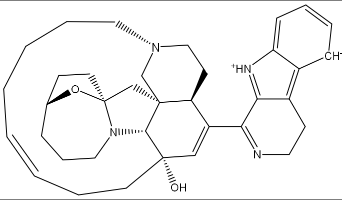	-10.98
CMNPD _18363	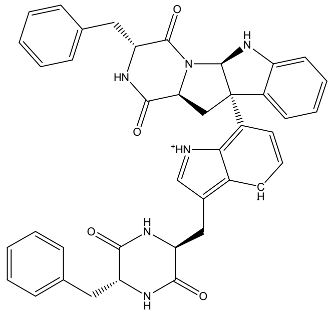	-11.688
CMNPD _20213	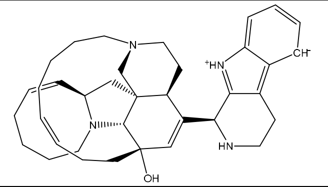	-11.297
**CMNPD _22964**	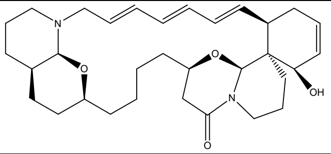	**-11.935**
CMNPD _22967	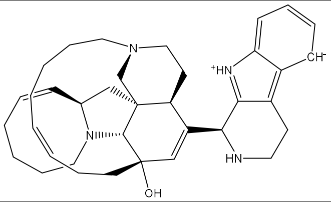	-11.322

Standard_F42202	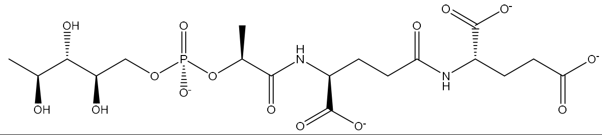	-6.385

Note: Bolded entries correspond to the three top-ranked hit candidates (Upenamide/CMNPD _22964 , Aspyronol/Compound_1749, and Fiscpropionate F/Compound_1796) selected for detailed molecular dynamics and MM-GBSA analyses.

Table S2. Predicted ADMET parameters of the top scored deep-sea compounds and standard using ADMET 3.0

**Compound ID**	**Molecular weight (g/mol)**	**Log P**	**HBD**	**HBA**	**TPSA (Å^2)**	**Pfizer rule**	**Lipinski`s rule**	**PBB %**
**Compound_1749**	**216.1**	**-0.201**	**2**	**5**	**75.99**	**Pass**	**Pass**	**21.1**
Compound_1665	391.23	4.54	3	5	73.99	Fail	Pass	91.1
Compound_1808	443.45	1.308	2	9	105.56	Pass	Pass	76.6
Compound_1794	473.35	3.935	2	5	77.84	Pass	Pass	71.3
Compound_1809	358.14	2.165	2	6	79.78	Pass	Pass	90.4
Compound_1540	481.25	2.664	3	7	111.69	Pass	Pass	85.8
Compound_1786	321.15	2.921	3	5	81.51	Pass	Pass	92.8
**Compound_1796**	**355.27**	**2.895**	**3**	**5**	**86.63**	**Pass**	**Pass**	**49.6**
Compound_1552	696.05	4.764	2	10	137.8	Pass	Pass	97.6
Compound_1799	386.14	3.39	3	7	113.29	Pass	Pass	98.3
Compound_1539	523.26	1.884	2	8	117.76	Pass	Pass	88.9

Compound_1529	238.19	3.078	2	2	40.46	Fail	Pass	67.7
Compound_1745	202.08	-0.632	3	5	86.99	Pass	Pass	11.2
Compound_1639	350.08	2.364	5	6	118.22	Pass	Pass	92.9
Compound_1757	310.1	1.762	2	6	82.17	Pass	Pass	74.3
Compound_1744	170.09	0.308	1	3	46.53	Pass	Pass	53.4
Compound_1624	278.08	0.558	4	6	107.22	Pass	Pass	85.8
Compound_1522	196.07	0.152	2	4	70.67	Pass	Pass	64.7
Compound_1816	368.27	5.036	1	2	37.3	Fail	Pass	94.7
Compound_1655	231.09	2.272	3	4	73.32	Pass	Pass	88.2
Compound_1763	348.12	1.247	2	7	102.29	Pass	Pass	93.3
Compound_1654	396.18	2.069	4	8	125.68	Pass	Pass	84.8
Compound_1802	459.15	1.046	3	10	125.79	Pass	Pass	57.1
CMNPD_14553	568.38	3.076	3	6	71.33	Fail	Pass	83.7
CMNPD_16909	566.36	3.418	1	6	60.66	Fail	Pass	89.8
CMNPD_18363	664.28	3.434	4	10	132.0	Pass	Pass	97.4
CMNPD_20213	552.38	3.597	2	5	51.1	Fail	Pass	88.9
**CMNPD_22964**	**522.35**	**2.802**	**1**	**6**	**62.24**	**Fail**	**Pass**	**67.2**

CMNPD _22967	552.38	3.597	2	5	51.1	Fail	Pass	88.9
Standard_F42202	546.15	-1.674	9	17	286.55	Pass	Fail	29

Note: Bolded entries correspond to the three top-ranked hit candidates (Upenamide/CMNPD _22964 , Aspyronol/Compound_1749, and Fiscpropionate F/Compound_1796) selected for detailed molecular dynamics and MM-GBSA analyses.
